# Identifying Opportunities and Challenges for Patients With Sarcoma as a Result of Comprehensive Genomic Profiling of Sarcoma Specimens

**DOI:** 10.1200/PO.19.00227

**Published:** 2020-03-18

**Authors:** Margaret A. Hay, Eric A. Severson, Vincent A. Miller, David A. Liebner, Jo-Anne Vergilio, Sherri Z. Millis, James L. Chen

**Affiliations:** ^1^ The Ohio State University, Columbus, OH; ^2^ Foundation Medicine Inc., Morrisville, NC; ^3^ Foundation Medicine Inc., Cambridge, MA

## Abstract

**PURPOSE:**

Comprehensive genomic profiling (CGP) of sarcomas is rapidly being integrated into routine clinical care to help refine diagnosis and prognosis and determine treatment. However, little is known about barriers to successful CGP or its clinical utility in sarcoma. We set out to determine whether CGP alters physician treatment decision-making, and whether sarcoma subtypes influence the frequency of successful technical performance of CGP.

**METHODS:**

A single-institution study evaluated profiling outcomes of 392 samples from patients with sarcoma, using a commercially available CGP panel. Of this group, 34 patients were evaluated prospectively (Decision Impact Trial) to evaluate the utility of CGP in physician decision-making. All cases were retrospectively analyzed to identify causes of CGP failure.

**RESULTS:**

CGP successfully interrogated 75.3% (n = 295 of 392) of patients with sarcoma. Bone sarcomas had lower passing rates at 65.3% (n = 32 of 49) compared with soft tissue sarcomas at 76.7% (n = 263 of 343; *P* = .0008). Biopsy location also correlated with profiling efficiency. Bone biopsy specimens had a 52.8% (n = 19 of 36) passing rate versus lung (61.1%; n = 33 of 54) and abdomen (80.1%; n = 109 of 136) specimens. CGP altered physician treatment selection in 25% of evaluable patients (n = 7 of 28) and was associated with improved progression-free survival.

**CONCLUSION:**

To our knowledge, this is the largest technical evaluation of the performance of CGP in sarcoma. CGP was effectively performed in the vast majority of sarcoma samples and altered physician treatment selection. Tumor location and tissue subtype were key determinants of profiling success and associated with preanalytic variables that affect DNA and RNA quality. These results support standardized biopsy collection protocols to improve profiling outcomes.

## INTRODUCTION

Sarcomas are uncommon, highly morbid cancers that constitute approximately 1% of all adult malignancies. Currently, more than 70 recognized histologic subtypes exist, which makes them extremely challenging to accurately diagnosis and treat.^1^ Sarcomas have multiple genomic alterations, including copy number changes, point mutations, insertions and deletions, and fusions.^2-4^ Thus, detailed RNA and DNA sequence analysis is key for diagnosis as well as for treatment decision-making.^5^

It is not surprising, therefore, that comprehensive genomic profiling (CGP) is increasingly being used in the evaluation and management of bone and soft tissue sarcomas. CGP is the sequencing of DNA and RNA from tumor samples, which enables the identification of known and novel alterations that may drive oncogenicity.^6-9^ CGP may refine the histologic tumor diagnosis, with its inherent ramifications for management.^10,11^ Indeed, in a study by Groisberg et al^1^ specific to sarcomas, the authors found 61% of 102 patients in a retrospective cohort had a potentially actionable genomic target_._

Despite the potential role that CGP may have in the treatment of sarcoma, the impact of DNA- and RNA-based CGP on physician decision-making is poorly documented.^12,13^ Similarly, it is equally unclear what factors may contribute to the success or failure of CGP in an outpatient clinical setting. Previous studies have assessed failure rates related to sarcoma tissue sample size (block *v* slide *v* core biopsy specimen), quality, and sample fixation methods.^14-16^ To our knowledge, the contribution of the various sarcoma subtypes to the success of CGP technical performance, particularly given the necessity for both DNA and RNA sequencing as mentioned previously is unknown; neither are the relative contributions of biopsy site (bone *v* soft tissue) and methods (ie, computed tomography [CT]-guided core needle biopsy *v* open excisional biopsy). To answer these questions, we herein report the results of two studies: The first is a prospective clinical trial examining the impact of DNA- and RNA-based CGP on physician decision-making. The second is a retrospective analysis of the success and failure rates of CGP in an even larger patient cohort, which includes the aforementioned patients.

## METHODS

### Sarcoma Decision Impact Clinical Trial

#### Study design.

To examine the effects of CGP on physician decision-making, a prospective clinical trial (The Ohio State University [OSU] Institutional Review Board approval no. OSU-12067) was performed in collaboration with Foundation Medicine. From 2014 to 2016, patients with sarcoma seen at OSU were approached to be consented for CGP. The primary objectives of the study were to assess the feasibility and logistics associated with a clinical trial using a commercially available CGP platform (FoundationOneHeme; Foundation Medicine, Cambridge, MA) in an academic clinical setting and to determine the proportion of patients who would receive a cancer-related therapy based on CGP results. Patients could enter this study at any line of therapy and must have had a tumor sample available for CGP testing. Patients also must have been within 10 weeks of starting their current line of therapy and enrolled before their next imaging appointment. Expected survival must have been > 3 months, as estimated by the treating oncologist. Full inclusion and exclusion criteria are presented in the Data Supplement. Our targeted enrollment was 40 patients, half with a diagnosis of a lipomatous tumor and half diagnosed with a nonlipomatous tumor.

#### Treatment plan.

Patients enrolled on the Decision Impact trial continued to receive investigator’s choice of therapy until time of progression as defined by clinical or radiographic criteria. Physicians determined the subsequent line of therapy without CGP results and then subsequently with the CGP results. If the treatment recommendation changed, this was considered a change in therapy. Either outcome was documented.

#### Statistical considerations.

Descriptive statistics were used to summarize the data. Treatment “switches” were noted and included in the overall analysis. Progression-free survival (PFS) was summarized using Kaplan-Meier survival analysis.

### The Ohio State Retrospective Sarcoma Study Data

#### Study design.

A retrospective study at OSU was performed using the medical center’s electronic health record (OSU Institutional Review Board approval no. 2016C0113). Specific information from patients who had sarcoma tumor samples sent for CGP was collected and included the following: date of sample procurement, method of specimen procurement (eg, excisional biopsy, needle core biopsy, fine needle aspiration), biopsy site, specimen source (hospital), and pathology classification by OSU. The Foundation Medicine records were interrogated for all sarcoma samples sent by OSU and the following information was compiled: specimen type (formalin-fixed paraffin-embedded tissue blocks *v* unstained slides), morphologic tumor purity, DNA and RNA extraction yield, sequencing metrics, and the nature of the released report (ie, pass, fail, or qualified). A qualified report indicates a sample for which it was unable to complete the entire CGP process (eg, RNA extraction, low tumor purity) but for which some genomic information was obtained. In contrast, a passed report indicates a sample for which DNA and RNA profiling was completed successfully. From this information, frequency and passing rates of sarcoma subtypes, biopsy locations, and biopsy methods were calculated. Frequency of sample failure from DNA and RNA failures was also evaluated.

#### Statistical considerations.

Descriptive statistics were used to summarize the data. χ^2^ analysis was used to calculate the significance of intergroup alterations.

## RESULTS

### Physician Decision-Making Was Prospectively Altered for 25% of Patients With Sarcoma Who Underwent CGP

**FIG 1.**
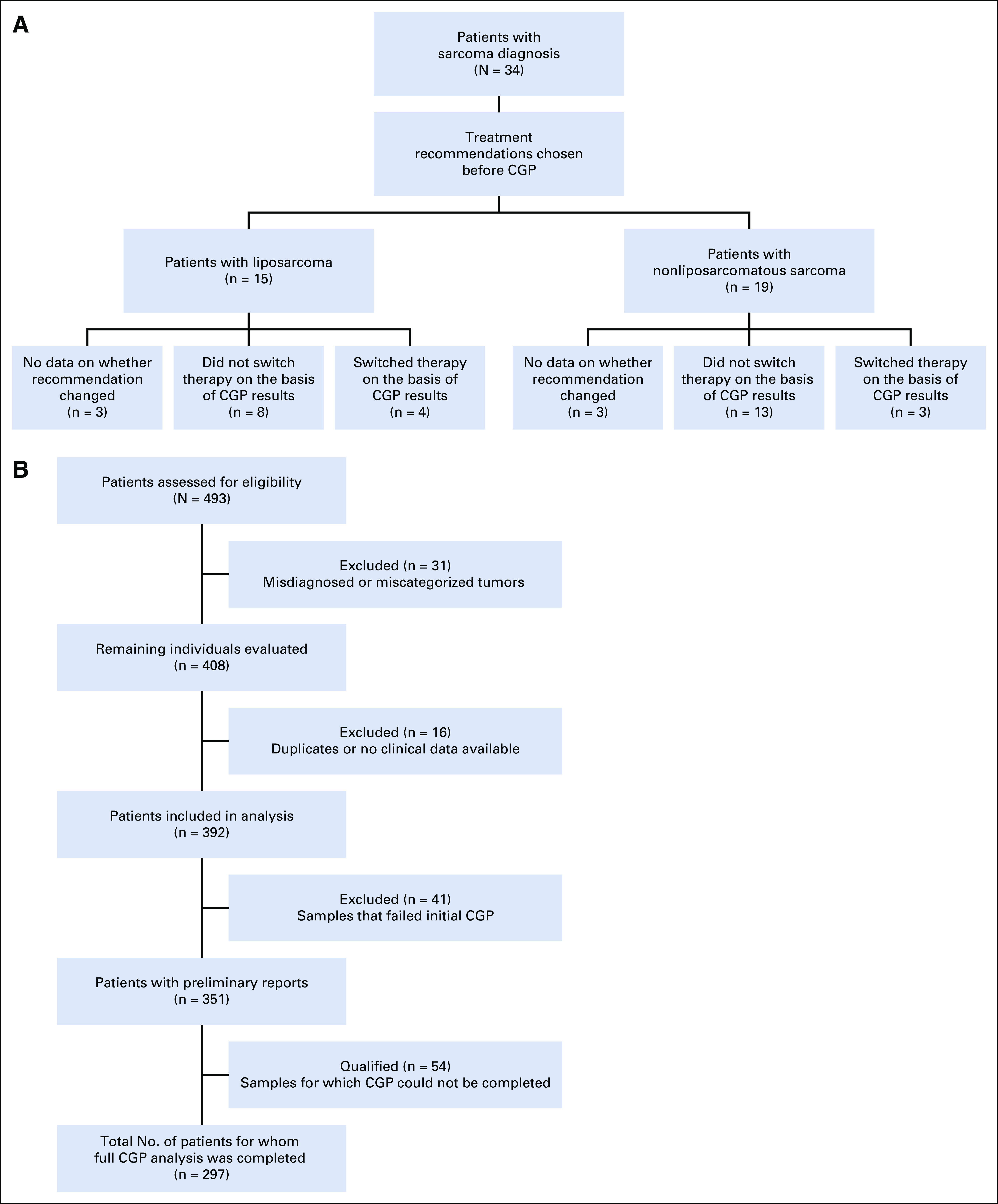
(A) Flow diagram of the Decision Impact Trial. The 34 patients diagnosed with sarcoma were categorized by the lipomatous or nonlipomatous nature of their tumor. For many patients, the recommended treatment did not change. In the lipomatous group, the recommended therapy for four patients changed on the basis of CGP results. In the nonlipomatous group, the recommended treatment changed for three patients. (B) Flow diagram for retrospective CGP failure analysis. A total of 439 patients were assessed for eligibility, of whom 31 were excluded when their diagnosis was altered by CGP and they were determined not to have a sarcoma. Of the remaining 408 patients, an additional 16 were excluded. This was partially due to finding duplicate patients (ie, some data were obtained with patients’ identities removed, because they were included in the Decision Impact Trial, which was blinded; in unblinding, we found some individuals had been included twice). Other reasons included being unable to locate individuals within IHIS (Ohio State University’s electronic medical record) and data necessary for additional analysis (including biopsy location and method) were unavailable. A total of 392 patients’ results were included in the analysis, from which there were 413 unique tumor biopsy samples. Of these, 41 individual patient samples failed initial CGP analysis; from these 41 patients, there a total of 44 unique samples failed testing. Initial samples (n = 56) from 54 patients were unable to complete full CGP testing. The remaining 295 individual patient samples passed initial CGP analysis. CGP, comprehensive genomic profiling.

As part of a Decision Impact Trial, patients with sarcoma whose disease had not yet progressed on the current line of therapy were approached to undergo CGP as part of the study. Treating oncologists were blinded to CGP results until time of progression (based on imaging results). Treating oncologists first documented their treatment decision for the patient with sarcoma without CGP results. CGP data were then released for review and the treating oncologist would note whether their treatment decision had been altered by the CGP results. Of the 34 patients enrolled in the study, 28 were evaluable. Of the six excluded patients, three patients were lost to follow-up and three patients had rapid disease progression precluding effective use of any secondary agent. A diagram indicating the patient groups in the trial and resulting treatment decisions after CGP is provided in Figure 1A. Of the 28 evaluable patients, 50% were male, median age was 62 years, and participant ages ranged from 24 to 80 years (Table 1).

**TABLE 1.**
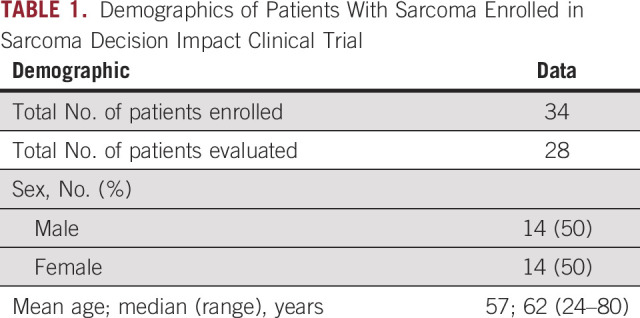
Demographics of Patients With Sarcoma Enrolled in Sarcoma Decision Impact Clinical Trial

Once the CGP results were revealed, 25% of patients (n = 7 of 28) had their treatments switched (Data Supplement). Detailed patient information on histology, molecular abnormalities, and the revised treatment, if applicable, is available in the Data Supplement.

**FIG 2.**
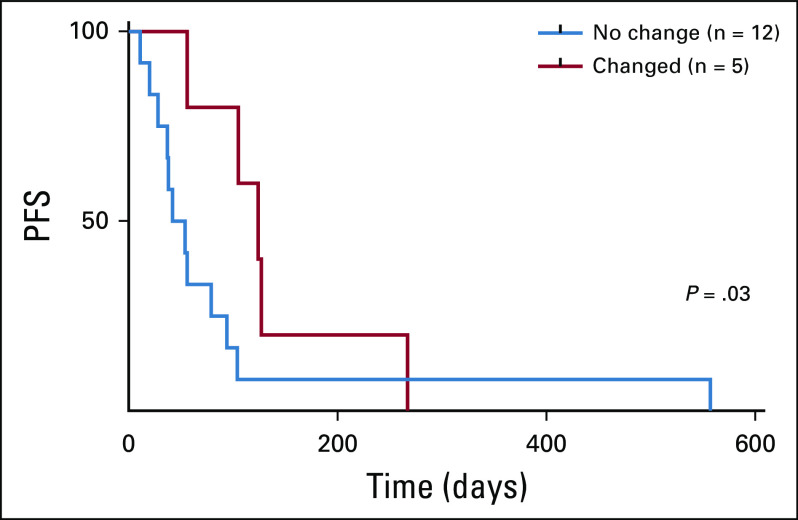
Effect of genomically informed treatments on sarcoma patients. Patients whose treatments were altered by CGP were significantly more likely to have a longer PFS than patients whose treatments were not changed (Wilcoxon *P* = .03). CGP, comprehensive genomic profiling; PFS, progression-free survival.

As part of our secondary analysis, we evaluated whether genomic-directed therapy may alter the overall PFS of patients with sarcoma. This analysis was performed with the caveat that sarcoma subtypes and availability may be confounders. Of the seven patients for whom treatment was altered, six patients received the selected treatment; one patient died before initiating therapy. As an exploratory end point, we noted that the median PFS in the CGP-selected group was 124 days versus 54 days in the non-CGP–selection group (*P* = .03; Fig 2).

### CGP Was Performed on Almost 400 Patients With Sarcoma

A total of 392 patients representing 413 unique samples were profiled over 3 years. Certain patients required multiple samples for processing, given initial failures; thus, the number of unique samples was greater than the number of individual patients. Demographics of these individuals are listed in Table 2 and the disposition of these patients is represented in Figure 1B. Just more than half of these patients (53%) were female, and the median age was 60 years (range, 16 to 89). Complete DNA and RNA sequencing results were reported for 76% (n = 313 of 413) unique tumor samples. More than 1,300 alterations were detected in the 413 unique samples, of which the most common are listed in the Data Supplement. L-type sarcomas (ie, leiomyosarcoma, liposarcoma) constituted the greatest proportion of cases, and this is indicative by the preponderance of TP53, RB1 alterations and MDM2 and CDK4 amplifications (Table 3).

**TABLE 2.**
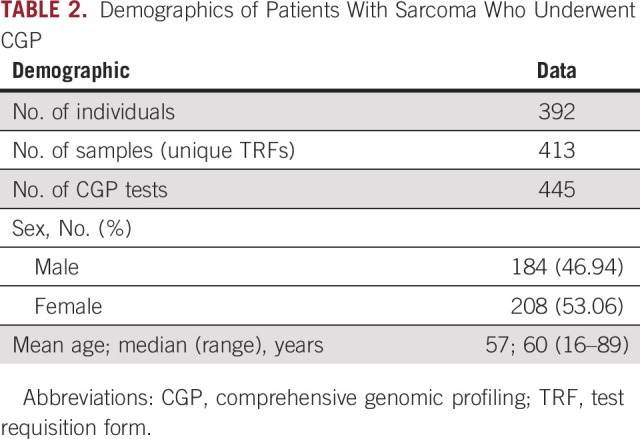
Demographics of Patients With Sarcoma Who Underwent CGP

**TABLE 3.**
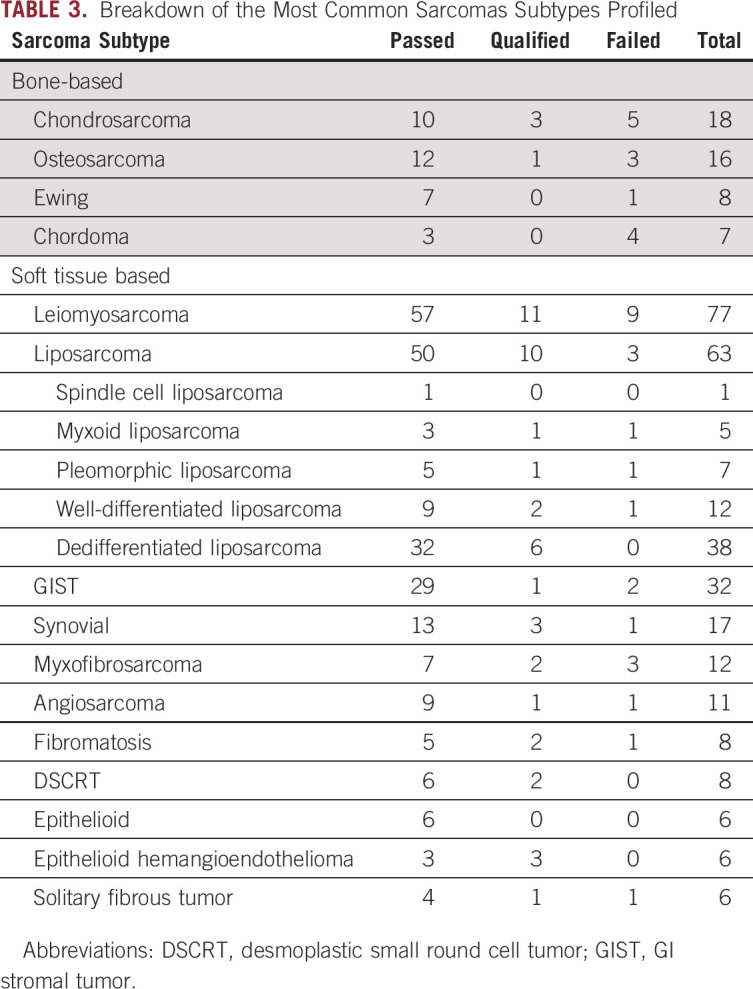
Breakdown of the Most Common Sarcomas Subtypes Profiled

**FIG 3.**
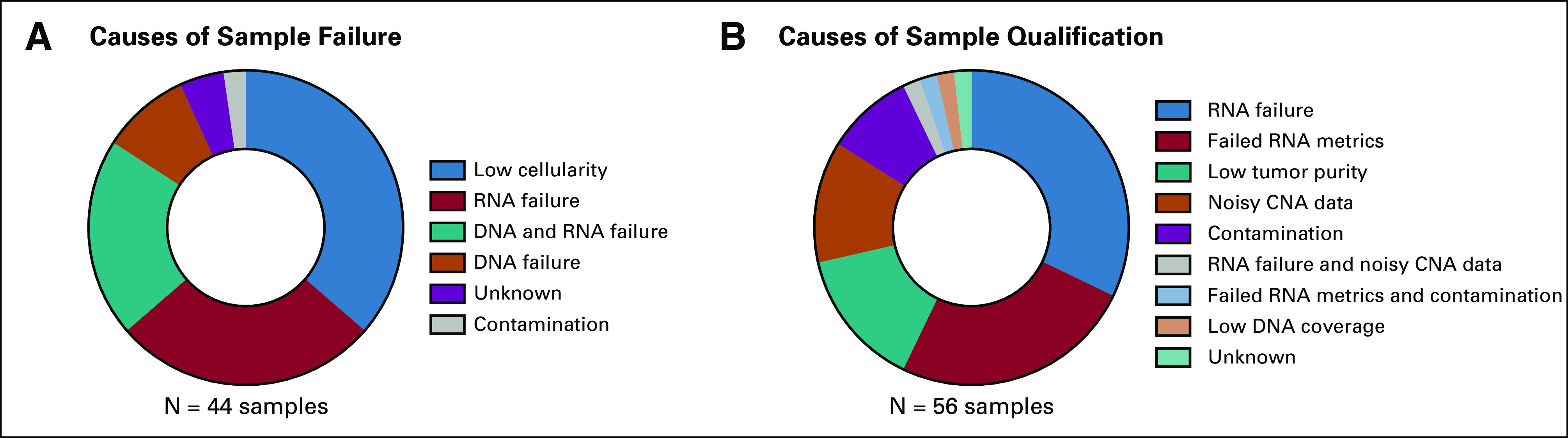
(A) The breakdown of causes of sample comprehensive genomic profiling (CGP) failure; the largest factor was low sample cellularity. This was followed by RNA failure, and then both DNA and RNA failure, with those three causes accounting for > 75% of failures. The remaining causes of sample failure were DNA failure, contamination, and unknown causes. (B) Breakdown of reasons samples did not completely pass CGP analysis, and thus were termed “qualified.” RNA failure, followed by failed RNA metrics, low tumor purity, noisy CNA data, and contamination accounted for > 90% the causes of incomplete CGP analysis. CNA, copy number alteration.

Fifty-six of the 413 samples (13.6%) had reports qualified for low tumor purity, failed RNA extraction, or suboptimal sequencing metrics, whereas 10.7% (n = 44 of 413) samples failed testing. Of failed samples, DNA failures made up 9.1% (n = 4 of 44), RNA failures 27.3% (n = 12), and DNA and RNA failure occurred in 20.5% (n = 9) of cases, as shown in Figure 3A. Of the qualified samples (n = 56), RNA failure (32.1%; n = 18), failed RNA metrics (25%; n =14), low tumor purity (14.3%; n =8), noisy copy number alteration data (12.5%; n =7), and contamination (8.9%; n =5) composed > 90% of the causes of incomplete sample CGP (Fig 3B).

### Sarcoma Originating From the Bone Had the Lowest CGP Pass Rate

Compared with other subtypes, bone sarcomas had statistically lower CGP passing rates of 65.3% (n = 32 of 49) than soft tissue sarcomas (76.7%; n = 263 of 343; χ^2^ *P* = .0008). Examining sarcoma subtypes individually, leiomyosarcomas (74.0%; n = 57 of 77) and liposarcomas (79.4%; n = 50 of 63) had proportionally higher pass rates than did bone-based sarcomas. Just over half of chondrosarcomas passed, and over one-quarter failed initial CGP. Chordomas had the lowest successful initial genomic profiling rate, with a 43% initial pass rate (Table 3).

### Biopsy Site and Method Influence Successful CGP Completion

#### Biopsy sites.

Biopsy sites were classified as follows: abdomen, bone, extremity, lung, liver, uterus, skin, soft tissue, and other. The “other” category encompassed an array of sites, including, but not limited to, brain, eye, epicardium, aorta, lymph node, submandibular gland, bladder, prostate, testes, ovary, cervix, and vagina. The most common biopsy sites represented were abdomen (n = 136) and soft tissue (n = 93). Bone biopsy specimens had the lowest pass rate for CGP profiling (52.8%; n = 19 of 36). Lung had the next lowest pass rate (61.1%; n = 33 of 54). Abdomen (including liver) was the most frequently biopsied site and had the highest pass rate: 80.1% (n = 109 of 136).

#### Biopsy method.

The most common biopsy methods were excisional, followed by CT-guided and ultrasound-guided biopsies. Excisional biopsy specimens had the highest pass rate (80%; n = 200 of 250), followed by specimens from US-guided biopsies (69.4%; n = 25 of 36) and then specimens from CT-guided biopsies (62.5%; n = 45 of 72).

In conclusion, to our knowledge, this study is the only prospective evaluation of DNA- and RNA-based CGP in sarcoma on physician decision-making and the largest in-depth evaluation of success of CGP in sarcoma to date. We note in this cohort that CGP in sarcoma is successful in a large majority of patients and alters physician treatment decision-making in an estimated 25% of patients. However, this is a small sample set. Nevertheless, we did note a difference in PFS. Furthermore, this percentage may have been driven by the fact that the study data were from the era when CDK4 inhibitors were just starting to be considered for use off-label in adipocytic tumors, and confirmation of CDK4 amplification was critical for this drug selection. We would anticipate, as time progresses, the types of tumor for which there are defined targetable alterations will increase and thus there would be an increase in physician treatment-decision changes. A classic example would be that of GI tumors for which KIT/PDGFR sequencing is now considered standard of care for determining whether a patient has these alterations and, if so, whether the patient has resistance alterations.^17^ A developing example would be that in leiomyosarcoma, where we have previously reported that homologous recombination alterations are a common feature of uterine leiomyosarcomas and may be amenable to PARP inhibition.^18^

Clearly, tumor location and tissue subtype significantly influence profiling success, likely secondary to preanalytic variables that influence quality of DNA and RNA, such as decalcification, tumor cellularity, and available tissue volume. Our results support implementing standard biopsy collection protocols for bone-based sarcoma (Data Supplement). Bone samples often undergo a decalcification process, which may destroy DNA and RNA, thus explaining their lower success rates. Of note, of the 36 bone biopsy samples that were collected in this study, pathology reports for only 13 mentioned the sample underwent decalcification; however, no specifications regarding the decalcification agent used or the fixation times were documented in the report. The pathology reports for the other 23 samples did not indicate whether decalcification was completed. Of note, of the 13 which did report decalcification, only five samples passed initial CGP. Given the significantly higher failure rate of bone biopsy samples with CGP, it is reasonable to infer that this is one of the important outcomes. Protocols to prevent decalcification during sample processing may aid in yielding higher CGP success rates.^19,20^ In addition, obtaining excisional biopsy specimens rather than imaged-guided biopsy specimens seems to lead to greater likelihood of CGP success.

In these studies, it should be mentioned that germline aberrations were not evaluated, because Foundation Medicine does not test for these. Furthermore, the patients included in this study were not tested for circulating free DNA, because we did not send blood samples, only tissue. These can be considered limitations of the study. In addition, another major limitation was pathology sample processing information. Regarding bone samples sent for CGP analysis, pathology reports would often indicate that decalcification was performed, but no additional details were provided. There was no documentation regarding decalcification agents used or duration of decalcification process. Also, many of our samples are often obtained at outside hospitals, and there is no standardization of pathology report contents.

In summary, sarcoma CGP appears to alter physician decision-making regarding treatment and may affect the ultimate outcome of the patient. Careful sample acquisition with attention to nucleic acid handling will be key to ensuring usable results.
